# Identification of Australian Aboriginal and Torres Strait Islander Cancer Patients in the Primary Health Care Setting

**DOI:** 10.3389/fpubh.2017.00199

**Published:** 2017-08-07

**Authors:** Audra de Witt, Frances C. Cunningham, Ross Bailie, Christina M. Bernardes, Veronica Matthews, Brian Arley, Judith A. Meiklejohn, Gail Garvey, Jon Adams, Jennifer H. Martin, Euan T. Walpole, Daniel Williamson, Patricia C. Valery

**Affiliations:** ^1^Menzies School of Health Research, Brisbane, QLD, Australia; ^2^Charles Darwin University, Darwin, NT, Australia; ^3^QIMR Berghofer Medical Research Institute, Brisbane, QLD, Australia; ^4^University Centre for Rural Health, University of Sydney, Sydney, NSW, Australia; ^5^Faculty of Health, University of Technology Sydney, Sydney, NSW, Australia; ^6^School of Medicine and Public Health, University of Newcastle, Callaghan, NSW, Australia; ^7^Southside Clinical School, University of Queensland, Brisbane, QLD, Australia; ^8^Princess Alexandra Hospital, Brisbane, QLD, Australia; ^9^Metro South Health Hospital and Health Service, Woolloongabba, QLD, Australia; ^10^University of Queensland, Brisbane, QLD, Australia; ^11^Aboriginal and Torres Strait Islander Health Unit, Queensland Health, Brisbane, QLD, Australia

**Keywords:** aboriginal health, identification of Indigenous cancer patients, primary health care, cancer, electronic patient records

## Abstract

**Background:**

Aboriginal and Torres Strait Islander Australians have poorer cancer outcomes and experience 30% higher mortality rates compared to non-Indigenous Australians. Primary health care (PHC) services are increasingly being recognized as pivotal in improving Indigenous cancer patient outcomes. It is currently unknown whether patient information systems and practices in PHC settings accurately record Indigenous and cancer status. Being able to identify Indigenous cancer patients accessing services in PHC settings is the first step in improving outcomes.

**Methods:**

Aboriginal Medical Centres, mainstream (non-Indigenous specific), and government-operated centers in Queensland were contacted and data were collected by telephone during the period from 2014 to 2016. Participants were asked to (i) identify the number of patients diagnosed with cancer attending the service in the previous year; (ii) identify the Indigenous status of these patients and if this information was available; and (iii) advise how this information was obtained.

**Results:**

Ten primary health care centers (PHCCs) across Queensland participated in this study. Four centers were located in regional areas, three in remote areas and three in major cities. All participating centers reported ability to identify Indigenous cancer patients attending their service and utilizing electronic Patient Care Information Systems (PCIS) to manage their records; however, not all centers were able to identify Indigenous cancer patients in this way. Indigenous cancer patients were identified by PHCCs using PCIS (*n* = 8), searching paper records (*n* = 1), and combination of PCIS and staff recall (*n* = 1). Six different types of PCIS were being utilized by participating centers. There was no standardized way to identify Indigenous cancer patients across centers. Health service information systems, search functions and capacities of systems, and staff skill in extracting data using PCIS varied between centers.

**Conclusion:**

It is crucial to be able to easily identify Indigenous cancer patients accessing health services in the PHC setting to monitor progress, improve and evaluate care, and ultimately improve Indigenous cancer outcomes. It is also important for PHC staff to receive adequate training and support to utilize PCISs efficiently and effectively.

## Introduction

Aboriginal and Torres Strait Islander Australians (respectfully referred to hereafter as Indigenous Australians), who make up 3% of the total Australian population and 4.3% in Queensland, do not share the same high standard of health experienced by other Australians (1). Cancer is one of the major contributors to these health inequities between Indigenous and non-Indigenous peoples (2) and is the second leading cause of death for Indigenous Australians (3). Indigenous Australians have poorer cancer outcomes compared to their non-Indigenous counterparts (4) and experience 30% higher cancer mortality rates (5). The reasons underlying these disparities are not fully explained but may be due to advanced cancer at diagnosis (6), reduced uptake of services and access to treatment (6), experiences of higher rates of comorbidities (6), and language barriers among Indigenous Australians (7). Health service-related factors, such as poor accessibility to health services, long waiting periods, low numbers of Indigenous professionals, and high staff turnover, also contribute toward poor cancer outcomes for Indigenous patients (8). In the state of Queensland, more than half of the Indigenous population lives outside metropolitan areas (4). When diagnosed with cancer, this is a challenge as many Indigenous Australians need to travel long distances to access cancer treatment as comprehensive treatment may not be available locally (9).

Primary care providers are increasingly playing an important role in the care and management of cancer patients, especially outside urban areas, and as a result, there are calls for increased primary care involvement in cancer management and follow-up across the cancer spectrum (10–15). Indigenous Australians have the option to utilize mainstream health services (e.g., general practitioners) and/or Indigenous-specific primary health care (PHC) services outside the hospital [e.g., Aboriginal Community Controlled Health Services (ACCHS)] where available. ACCHS provision includes health promotion as well as diagnostic and treatment-based services for a wide range of health conditions in various settings. ACCHS and other Indigenous-specific services (provided through hospitals, community settings, and other health care facilities) were established in recognition of the importance of providing culturally appropriate and accessible health care services. Funding for ACCHS can be complex and they are funded by the Australian federal and/or state and territory governments (16).

Many Indigenous cancer patients in Australia are not identified as Indigenous in cancer registries (17). The accuracy of Indigenous identification in Australian public hospital admission records was estimated to be 88% correctly identified in 2011–2012 (18). However, it is not known whether current Patient Care Information Systems (PCIS) and practices in the PHC setting accurately record Indigenous status and cancer details (e.g., diagnosis, staging and cancer treatment plans) and there has been limited research in this area (19). Hence, the objective of this paper is to examine the systems and processes in place to identify Indigenous cancer patients accessing PHC services as a step toward improving Indigenous cancer outcomes. The study will also provide baseline information to assist with making recommendations for improvements. This study is a novel exploratory assessment of the patterns of care of Indigenous cancer patients at the PHC setting in Queensland, Australia.

## Materials and Methods

### Study Design and Study Sites Selection

The data reported here were collected from a cross-sectional study of the patterns of care of Indigenous cancer patients at the PHC setting in Queensland, Australia. A non-random purposive convenience list of (i) ACCHS in Queensland (identified via the Queensland Aboriginal and Islander Health Council website); (ii) mainstream (not Indigenous-specific services); and (iii) Queensland Health-operated primary health care centers (PHCCs), which provide services to large numbers of Indigenous patients, were compiled by the research team. A mixture of PHCCs located in remote, rural, and urban areas, community controlled and Queensland Health-operated services were included in this list. The inclusion criteria for PHCCs required centers to have at least ten Indigenous cancer patients attending their service, or be an Indigenous Community Controlled PHCC. Centers that met the eligibility criteria and were willing to participate in the study were included in the patterns of care component of the study. This included a visit to the PHCC from the research team to review the medical charts of Indigenous cancer patients (data not described here).

### Procedure

All purposively selected PHCCs were approached via email by an Indigenous community engagement officer, who followed up by a telephone call. The officer collected information via telephone using a standardized script. Responses were transcribed verbatim. These interviews were conducted from 28th August 2014 to 6th May 2016. PHCC staff were asked whether their service was able to (i) identify the number of patients diagnosed with cancer that attended their service in the previous year using their patient record system and/or information systems; (ii) identify the Indigenous status of these patients and if this information was available; (iii) report how many Indigenous Australians attended the clinic in the past year; and (iv) how this information was obtained. PHCCs that had at least ten Indigenous cancer patients attending their service, or was an Indigenous-specific PHCC, were invited to take part in this study. PHCCs included in the study were asked to prepare a list of all Indigenous cancer patients seen at their service. Patient eligibility included the following: age 18 years or over; be identified as Aboriginal or Torres Strait Islander; have a cancer diagnosis after 2010; and to be considered by service as “active patients” (currently accessing the PHCC). In a visit to the PHCC, the research team members received a brief training session from PHCC staff on their local PCIS. With the permission of PHCC staff, a member of the research team conducted additional searches of the PHCC’s database to identify other (if any) potentially eligible patients.

### Data Analysis

Data were collated and managed in an electronic database using Microsoft Excel 2010. The incidental observations of the research team collecting data for the study were also compiled within the spreadsheet. A descriptive approach was utilized to synthesize and analyze the data. Basic descriptive details (total numbers, percentages, and averages) were reported.

Ethics approvals for the study were obtained from Human Research Ethics Committees of the Darling Downs Hospital and Health Service, Menzies School of Health Research, and QIMR Berghofer Medical Research Institute.

## Results

### Health Center Participants

**Figure 1:**
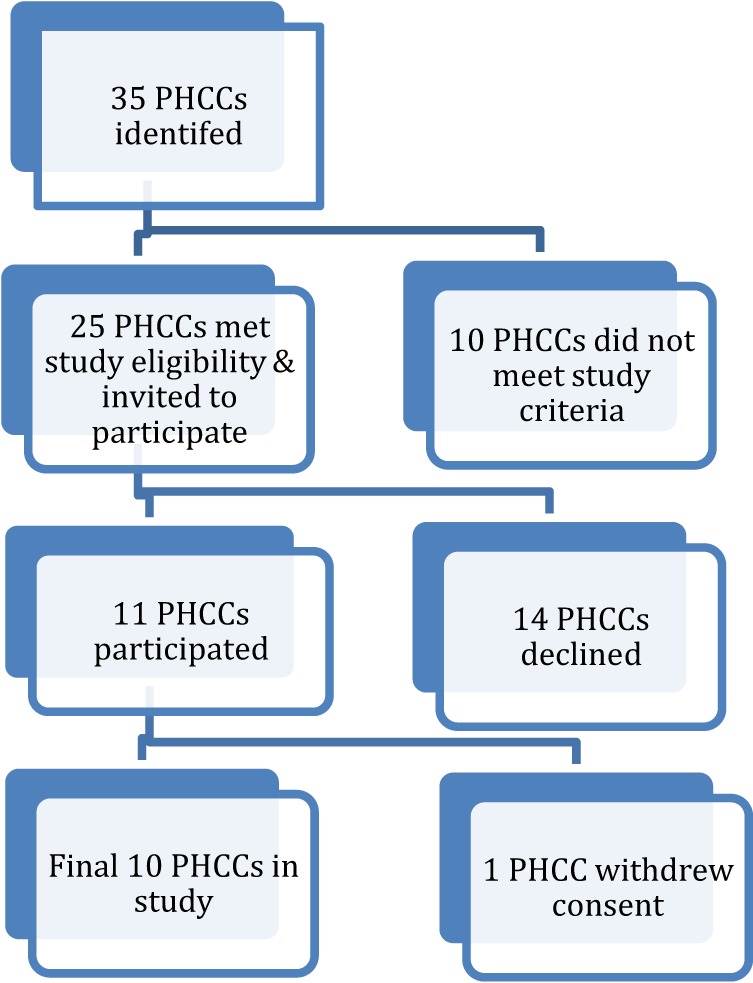
Primary health care center (PHCC) study recruitment process.

Thirty five PHCCs were approached and assessed against the eligibility criteria. Ten did not meet the study criteria and 25 were invited to participate. Of the 14 PHCCs that declined to participate, 12 were ACCHSs. PHCCs were located in a major city (*n* = 8), very remote areas (*n* = 5), and a remote location (*n* = 1). One of the 11 PHCCs that agreed to participate in the study later withdrew consent. See Figure 1 for PHCC study recruitment process.

Ten PHCCs across Queensland participated in this study. A diverse range of centers were represented geographically with four in regional areas, three in remote areas, and three in major cities. Nine of the center participants reported to be an ACCHS and one center reported to be a government-operated community center (Queensland Health). Information obtained for this study was from PHCC staff, namely practice managers (*n* = 5); quality assurance managers (*n* = 2); directors (*n* = 1); Indigenous health worker (*n* = 1); and a general practitioner (*n* = 1). Table 1 provides a summary of the participating health center characteristics.

**Table 1:** Primary health care center (PHCC) characteristics and number of Indigenous cancer patients identified.

Study site	Governance	Location	Patient information system	Total number of active patients (2016/2017)	Number of Indigenous cancer patients identified by PHCC staff	Eligible patients (confirmed by research staff)	Non-eligible patients		
							No cancer	Non-Indigenous	Other reasons
1	Indigenous Community Controlled Primary Health care (PHC)	Major city	Mmex	6,244^a^	39^e^	22	7	–	10
2	Indigenous Community Controlled PHC	Major city	Mmex	3,590^b^	66^f^	26	18	–	22
3	Indigenous Community Controlled PHC	Outer regional	Best practice	1,780	7^g^	7	–	–	–
4	Indigenous Community Controlled PHC	Outer regional	PenCat	5,409	30^e^	29	1	–	–
5	Queensland Health	Very remote	HCBIS + paper records	1,683^c^	6^h^	6	–	–	–
6	Indigenous Community Controlled PHC	Very remote	Best practice	1,546^d^	20^f^	12	3	–	5
7	Indigenous Community Controlled PHC	Inner regional	Communicare	4,150	221^i^	14	145	51	12
8	Indigenous Community Controlled PHC	Outer regional	Medical director	2,289	22^f^	21	–	1	–
9	Indigenous Community Controlled PHC	Remote	Medical director	2,000	8^f^	7	–	–	1
10	Indigenous Community Controlled PHC	Major city	PenCat	5,073	52^f^	14	11	3	24

*^a^Indigenous patients only*.

*^b^2,561 Indigenous patients*.

*^c^1,408 Indigenous patients*.

*^d^660 Indigenous patients*.

*^e^Research staff allowed to search PHC database and patient list provided by PHC staff*.

*^f^Research staff allowed to search PHC database*.

*^g^Research staff not allowed to search PHC database, and combination of electronic search and manual identification done by PHC staff*.

*^h^Research staff not allowed to search and manual identification by staff conducted (paper records)*.

*^i^Research team allowed to search using patient name only and patient list provided by staff*.

### Service Characteristics

Most of the centers (*n* = 8) reported on the total number of (Indigenous and non-Indigenous) active patients attending their center and three of these provided additional information on the number of Indigenous “active patients” (centers 2,5,6). In total, there were 33,764 active patients (Indigenous and non-Indigenous) across the ten participating centers. Only two centers provided a definition of active patients, that is, patients who have accessed health center services at least three times in the last 24 months. The largest center reported a total of 5,409 active patients (center 4) and, in contrast, the smallest center reported a total of 1,683 active patients (center 5). On average, the total number of active patients per service was 3,058 patients. One service only provided the number of Indigenous active patients rather than the total number of active patients attending the center (center 1). Of the active patients, 471 patients were identified by PHCCs to be potentially eligible for the study. Of these 471 patients identified as eligible by the PHCC, only 158 of patients were confirmed to be eligible according to the study inclusion criteria by the research team.

Six different types of electronic PCIS were reportedly being used by participating health centers. In this study, Medical Director (*n* = 2), Best Practice (*n* = 2), Mmex (*n* = 2), and PenCat (*n* = 2) systems were the most commonly utilized PCISs. Other PCISs included Communicare (*n* = 1) and the HBCIS (*n* = 1) system. (HBCIS reporting system is a type of PCIS that is commonly utilized by hospitals and this center is attached to a hospital). While all participating centers reported utilizing electronic patient information systems to manage their records, one center was only able to identify study participants by using paper records (*n* = 1), and another needed to use manual identification methods in addition to using the PCIS to identify Indigenous and cancer patients for the study.

The majority of health centers (*n* = 9) reported the ability to identify patients with a cancer only diagnosis attending their health service in the past year by searching their PCIS. However, all centers reported they were able to identify Indigenous patients with a cancer diagnosis searching their electronic records using the combination of both “Indigenous” and “cancer” search terms.

### Identification of Indigenous Cancer Patients

Indigenous cancer patients were identified at participating PHCCs in three different ways for study inclusion. Half of the centers (*n* = 5) allowed the research team to search the health center database to identify patients for the study, three centers provided a list of patients to the research team and also allowed the team to undertake further searches for eligible patients, and two centers provided the research team with a list of patients. However, it was not possible for the research staff to conduct further searches as one center was not able to identify Indigenous cancer patients using their electronic database; thus, manual identification by center staff was conducted using paper records. The other center did not allow the research team to conduct searches.

PHCCs identified Indigenous patients diagnosed with cancer at their service either by (i) the use of electronic medical records (*n* = 8); (ii) a combination of electronic records and staff knowledge (*n* = 1); or (iii) through manual identification (staff knowledge) (*n* = 1).

In addition to the above, services were also asked to estimate the number of Indigenous patients diagnosed with cancer who attended their health center in the previous year. The majority of centers (*n* = 9) reported that less than ten Indigenous cancer patients accessed their health center in the past year. Only one center (center 7) reported that more than 50 Indigenous cancer patients accessed their health center in the previous year. All PHCCs reported finding this information using a PCIS. However when centers were requested to provide further information, some variation in responses was identified. Eight centers reported that they were able to identify Indigenous status and cancer treatment records. One center reported that a combination of local knowledge and electronic data system was used to identify Indigenous status and cancer treatment records (remote location). Another center reported that their PCIS could identify cancer patients but not the Indigenous status of patients (inner regional location).

### Challenges in Patient Identification

The research team found that it was not a simple process to identify all potentially eligible patients for the study. There was no uniform and/or standard process of conducting searches on patient information systems across all participating PHCCs. Health service information systems, search functions and/or abilities of various systems, and staff knowledge on the use of the electronic systems to extract these data varied. For example, some systems had drop down boxes where cancer search terms could be selected, others did not have this capacity, and some had a combination of both. Examples of some search terms used by the research team to identify potentially eligible patients included: neoplasm, lymphoma, leukemia, breast tumor, carcinoma, prostate cancer, sarcoma, and adenocarcinoma. Furthermore, time constraints, physical space limitations, and the capacity of PHCC staff to provide database training to the research team posed challenges to more thorough patient ascertainment.

In addition, as seen in Table 1, searches conducted by PHCC staff and the research team produced varying results identifying Indigenous cancer patients for this study. For example, one center (center 7) using their PCIS search reported 221 eligible Indigenous cancer patients for the study when the research team only found 14 eligible patients (145 of the 221 patients were found not to have a cancer diagnosis, 51 patients were not Indigenous, and the remaining 12 patients were not eligible due to other reasons).

## Discussion

Findings from this study indicated that there was no systematic method for identifying Indigenous patients diagnosed with cancer who accessed health services in the PHC setting. Although all participating PHCC reported utilizing a form of PCIS (six different systems), not all centers were able to utilize the PCIS to identify Indigenous and cancer patients. It was noted that PHCC staff appeared to have varying skills, knowledge, and confidence in navigating the PCIS database. Database searches conducted separately by PHCC staff and the research team produced varying results in identifying Indigenous cancer patients for this study.

### No Standardized Method for Identifying Indigenous Cancer Patients

The principal reason to identify Indigenous cancer patients in the PHC setting is to enable better access to appropriate cancer care. In this study, there was no uniform method for identifying Indigenous patients diagnosed with cancer who accessed health services in the PHC setting by PHCC staff. The use of a number of different PCISs across centers, with varied functionalities and capacities, varying levels of staff ability and knowledge to utilize PCISs, and issues relating to manual identification of Indigenous cancer patients when electronic records were not utilized, all contributed to differences in methods of identifying Indigenous cancer patients in this study. Although all the centers in this study initially reported their ability to identify Indigenous cancer patients attending the PHCC using their electronic database, after further investigation (asking centers to provide a list of patient numbers and their cancer diagnosis), not all centers could find this information electronically.

This difficulty in extracting information from electronic databases has been reported in local and international literature where studies have found wide variations in accuracy and completeness of information stored in electronic patient records (20, 21). In regard to extracting records of patients diagnosed with cancer, one study identified a significant number of omissions in cancer diagnosis in the medical records of five general practices in the UK (22) and another study found that cancer diagnosis information that was recorded was less complete and detailed compared to what was recorded in the Regional Cancer Registry (23). In Australia, many Indigenous cancer patients are still not accurately identified in cancer registries and Indigenous patients are under-identified in general practice (24). Schutze and colleagues further suggest that under-identification of Indigenous cancer patients in GP settings in urban areas is related to a lack of staff understanding of its significance; inefficient record management systems to identify and/or record Indigenous patient status; and health centers failing to promote Indigenous status identification (24). Furthermore, although the collection and recording of Indigenous status is higher in the Australian ACCHS sector than in mainstream practice, Indigenous Australians may choose not to disclose their indigenous status due to fear of repercussions, such as anticipating a lower quality of service and racist attitudes after identifying (25).

### The Use of a Variety of PCIS

Six different PCISs were utilized by participating PHCCs in Queensland in this study. This variation of patient information systems utilized in the PHC setting poses a challenge when attempting to identify comparisons between services as each system varies in function capacities, capabilities, complexities, and “search-abilities.” PCISs vary in sophistication and due to system complexity may sometimes contain design flaws that can be difficult to detect (26, 27) and can sometimes generate new errors (28, 29) rather than preventing errors. Some of the identified problems in software packages include Indigenous status of patients not linked to clinical decision support (30); Indigenous status completion is non-mandatory (31); and Indigenous status is not included in GP-generated documentation (e.g., pathology requests) from which national data are collected (32). The lack of standardization and consistency of data items and appropriate data standards in PCISs and the lack of standardized methods to assess the quality of data in electronic records is a major challenge in the PHC setting as it makes it difficult to compare data between services and results across studies (33). One study that examined the consistency of denominator data extracted from electronic health records across PHC services in Australia found significant inconsistencies in denominator data. As these denominator data are used to calculate a range of national key performance indicators (nKPIs) across individual PHC services, questions arise about the reliability of nKPIs (21). In the hospital systems in Australia, there has been progress on the agreement and development of standardized data sets and systems. Similarly, the use of standardized data sets in the PHC setting would provide consistency of recording of data items across PHC services allowing for comparisons to be easily made across and between PHC services irrespective of local PCISs utilized.

The use of different PCISs can potentially also result in the fragmentation of care and service delivery. Another longer-term alternative to consider in addition to the standardization of reportable PHC data sets is the use of a standardized and/or compatible electronic system across the board that could result in potential benefits on many levels. The introduction of electronic health records is seen as a step toward a more effective and efficient health system as information sharing is one of the core foundations in providing high-quality care (34). Electronic health records can allow health care professionals to monitor and manage patients’ conditions. Information uploaded onto electronic health records using a secured online platform can also allow patients to keep track of their personal records, as well as providing a platform where patient information is shared between health providers (shared patient records with patient consent), promoting continuity of care. Patient information, such as medical history, prescribed medications, allergies, immunization history, and other personal information such as Indigenous status, are uploaded onto the system.

### Staff Skills, Knowledge, and Confidence in Navigating PCIS

The level of staff skills and knowledge in utilizing and navigating PCIS can contribute to the uncertainty of data accuracy and data reliability. This includes the ability of staff to accurately enter, manage, and extract Indigenous cancer patient information data using the local PCIS (21). Prior to visiting participating PHCCs, a number of PHCCs informed the research team that they were experiencing difficulties in extracting Indigenous patient cancer data from PCIS and needed more time to seek further assistance. During visits to participating centers, the research team noted that skills to navigate the PCIS varied between staff members at and between PHCCs. These observations are consistent with other research that identifies staff skills and confidence in use of electronic health systems as a significant limitation in the effective use of electronic health records (21, 35, 36). In situations where manual identification was required, the use of staff recall in identifying Indigenous patients accessing services at PHCCs was also limited by individual staff memory and staff turnover. These potential data reliability and staff knowledge issues are of concern as individual health centers need to be able to easily identify all Indigenous cancer patients accessing their service to be able to effectively monitor patient progress, outcomes, quality of care, and for quality improvement and service evaluation purposes.

In addition, as reported in the results, the searches conducted by PHCC staff and the research team to identify Indigenous cancer patients for the study produced varied results. Although the reasons for these differences in search results were not clearly evident, this may be due to variety of factors, such as health center staff having limited time and resources to undertake searches; staff overlooking study eligibility criteria; limited search terms; and staff experiencing difficulties (knowledge deficits) navigating the PCIS. The research team’s limited knowledge and experience utilizing PCIS databases may have also contributed to the variation in search results.

### Strengths and Limitations

This study investigates for the first time whether and how Indigenous cancer patients are identified at the PHC setting in Queensland, Australia. There are, however, some limitations to this study. Although the research team attempted to recruit a cross-section of PHCCs (e.g., remote, rural, and urban, community controlled and Queensland Health), the number of participating centers was small, purposively selected, and may not be representative of over 250 PHC services in Queensland. It is important to note that it is difficult to determine the exact number of PHCCs in Queensland as this varies depending on how PHC services are defined; therefore, numbers are indicative only (Queensland Health, unpublished data). In addition, there were inconsistencies in the quality of information, thus, there may be a need to interpret study results with some caution. However, the information in this study adds to the limited existing research and provides some important leads for further research.

### Implications for Policy, Practice, and Research

Improving the cancer outcomes for Indigenous Australians is a shared responsibility and requires commitment, collaboration, determination, and dedication by all stakeholders, including government, non-government, and community organizations. Improving Indigenous identification in cancer registries, hospitals, and in PHC settings is crucial in order to improve the health outcomes of Indigenous cancer patients. In the PHC setting, electronic patient information records across Queensland need to firstly be able to systematically identify Indigenous patients diagnosed with cancer in an efficient and effective manner. This paper provides some insight into the difficulties experienced by some PHCCs in identifying Indigenous cancer patients and is a starting point in which further investigation is required. Further research will be useful in reinforcing the significance of this, and will also provide guidance for policymakers toward the standardization of processes in identifying Indigenous cancer patients in the PHC setting.

It is imperative that all PHCCs are able to easily identify Indigenous patients diagnosed with cancer using consistent and standardized methods. It is important for PCISs to easily “flag” patients with a cancer diagnosis rather than using cumbersome approaches of having to search for specific cancer conditions separately. This could mean that all patients with a cancer diagnosis can be easily, accurately, and quickly identified. It is difficult to monitor progress, improve cancer care, and ultimately improve patient outcomes without being able to firstly identify cancer patients. Patient electronic databases vary in sophistication and complexity and software programs are regularly upgraded and updated. The formulation and use of standardized terms and datasets in the PHC setting for Indigenous patients diagnosed with cancer could be a way of effectively identifying, monitoring, and comparing patient outcomes within and between PHCCs. The appropriate use of secured electronic health records with patient consent has the potential benefit of improving the quality and continuity of patient care which ultimately leads to improved Indigenous cancer outcomes. However, it is important for all relevant health professionals to be provided with regular and adequate training in the accurate ascertainment and recording of Indigenous status and the use and functions of electronic databases to ensure proficient use, familiarity, and knowledge of utilized systems.

## Ethics Statement

Ethics approvals for the study were obtained from the Human Research Ethics Committees of the Darling Downs Hospital and Health Service, Menzies School of Research, and QIMR Berghofer Medical Research Institute. Participants were provided with informed consent and provided their written consent to participate in the study. All participants gave written consent in accordance with the Declaration of Helsinki.

## Author Contributions

PV, RB, GG, JHM, EW, JA, and DW designed the study. ADW, BA, CB, and JAM contributed with data acquisition. ADW, FC, CB, VM, JAM, and BA assisted with interpretation of data. ADW drafted the report and all authors revised it critically, contributed to its editing, and approved its final version.

## Conflict of Interest Statement

The authors declare that the research was conducted in the absence of any commercial or financial relationships that could be construed as a potential conflict of interest.
